# Immunohistochemical Investigation into Protein Expression Patterns of FOXO4, IRF8 and LEF1 in Canine Osteosarcoma

**DOI:** 10.3390/cancers16101945

**Published:** 2024-05-20

**Authors:** Simone de Brot, Jack Cobb, Aziza A. Alibhai, Jorja Jackson-Oxley, Maria Haque, Rodhan Patke, Anna E. Harris, Corinne L. Woodcock, Jennifer Lothion-Roy, Dhruvika Varun, Rachel Thompson, Claudia Gomes, Valentina Kubale, Mark D. Dunning, Jennie N. Jeyapalan, Nigel P. Mongan, Catrin S. Rutland

**Affiliations:** 1 School of Veterinary Medicine and Science, Faculty of Medicine and Health Sciences, University of Nottingham, Nottingham NG7 2RD, UK; simone.debrot@unibe.ch (S.d.B.); mzyjc18@exmail.nottingham.ac.uk (J.C.); svaaa3@exmail.nottingham.ac.uk (A.A.A.); svxjj1@exmail.nottingham.ac.uk (J.J.-O.); stxmh36@exmail.nottingham.ac.uk (M.H.); stxrp16@exmail.nottingham.ac.uk (R.P.); svzaeh@exmail.nottingham.ac.uk (A.E.H.); svzclw@exmail.nottingham.ac.uk (C.L.W.); svzjhl@exmail.nottingham.ac.uk (J.L.-R.); stxdv8@exmail.nottingham.ac.uk (D.V.); styrt5@exmail.nottingham.ac.uk (R.T.); svxcg4@exmail.nottingham.ac.uk (C.G.); mark.dunning@nottingham.ac.uk (M.D.D.); plzjnj@exmail.nottingham.ac.uk (J.N.J.); 2 Comparative Pathology Platform of the University of Bern (COMPATH), Institute of Animal Pathology, University of Bern, 3012 Bern, Switzerland; 3 Institute of Preclinical Sciences, Veterinary Faculty, University of Ljubljana, 1000 Ljubljana, Slovenia; valentina.kubaledvojmoc@vf.uni-lj.si; 4 Willows Veterinary Centre and Referral Service, Solihull B90 4NH, UK; 5 Faculty of Medicine and Health Science, Biodiscovery Institute, University of Nottingham, Nottingham NG7 2RD, UK; 6 Department of Pharmacology, Weill Cornell Medicine, New York, NY 10075, USA

**Keywords:** cancer identification, forkhead box protein O4, interferon regulatory factor 8, lymphoid enhancer binding factor 1, osteosarcoma, pathology

## Abstract

**Simple Summary:**

There have been limited advances in the diagnosis, prognosis, and treatment of both canine and human osteosarcoma (OSA), the most common type of primary bone cancer. OSA has an aggressive nature, with incidence rates ranging from 13.9 to 27.2 cases per 100,000 dogs, yet there have been limited advances in patient outcomes in recent decades. Recent developments have identified similarities between human and canine OSA; therefore, researching naturally occurring canine bone cancer may help inform research into OSA in people. The present research investigated three proteins, FOXO4, IRF8, and LEF1, to visualise their expression in OSA tissue. This research helps us understand where the proteins are being expressed in the tumours, which genetic pathways are changing, and may help us identify potentially informative diagnostic, prognostic, and treatment avenues for this cancer in dogs and people.

**Abstract:**

Osteosarcoma (OSA) is the most common type of primary bone malignancy in people and dogs. Our previous molecular comparisons of canine OSA against healthy bone resulted in the identification of differentially expressed protein-expressing genes (forkhead box protein O4 (*FOXO4*), interferon regulatory factor 8 (*IRF8*), and lymphoid enhancer binding factor 1 (*LEF1*)). Immunohistochemistry (IHC) and H-scoring provided semi-quantitative assessment of nuclear and cytoplasmic staining alongside qualitative data to contextualise staining (*n* = 26 patients). FOXO4 was expressed predominantly in the cytoplasm with significantly lower nuclear H-scores. IRF8 H-scores ranged from 0 to 3 throughout the cohort in the nucleus and cytoplasm. LEF1 was expressed in all patients with significantly lower cytoplasmic staining compared to nuclear. No sex or anatomical location differences were observed. While reduced levels of FOXO4 might indicate malignancy, the weak or absent protein expression limits its primary use as diagnostic tumour marker. IRF8 and LEF1 have more potential for prognostic and diagnostic uses and facilitate further understanding of their roles within their respective molecular pathways, including Wnt/beta-catenin/LEF1 signalling and differential regulation of tumour suppressor genes. Deeper understanding of the mechanisms involved in OSA are essential contributions towards the development of novel diagnostic, prognostic, and treatment options in human and veterinary medicine contexts.

## 1. Introduction

Osteosarcoma (OSA) is a neoplasia of mesenchymal origin, which tends to derive from the medullary cavity of metaphyseal bones and subsequently expands to the cortical bone; this pathological process is named central OSA [1,2]. Rarely, OSA can originate from the periosteal surface and is thought to be overall less aggressive compared to central OSA [2]. Canine OSA is considered the most common bone tumour identified, with a documented prevalence of approximately 85% of all primary malignancies arising in the skeleton of this species and 3–4% of all malignant tumours in dogs [3,4,5,6]. Reported OSA incidence is greater in canines than in any other species, with an estimated rate of 13.9–27.2 cases per 100,000 dogs [7,8,9,10]. OSA incidence in humans is much lower, at 0.89–1.2 per 100,000 [8,10,11]. This high incidence in dogs not only emphasises the veterinary challenge posed by OSA, but also enhances the efficacy of the canine model as the low incidence rate in humans is a big factor in the lack of understanding observed to date. The low incidence in people also contributes towards the lack of diagnosis and treatment options, and the relatively poor outcomes following OSA diagnosis.

Linking OSA incidence with specific risk factors can allude to the involvement of certain biological pathways. Canine OSA predominantly affects middle-aged, naturally larger breeds including Rottweilers, Great Danes, Saint Bernards, Doberman Pinschers, and Irish Wolfhounds [7,11,12,13]. In addition, increased body weight (even after controlling for breed), height, and age are risk factors for OSA in dogs [12]. These link with human OSA risk factors, as taller and heavier individuals are more prone to OSA formation [14,15,16]. This is further reinforced by higher incidence in males across both species, who on average naturally grow to be slightly larger [10,12,16,17]. These risk factors also implicate bone growth as potentially causative in OSA formation. The growth risk factor may also correspond to the respective risk of certain age groups. In people, OSA formation follows a bimodal trend with the primary peak being in adolescence, contributing to over 50% of cases. There is then another smaller peak in seniors [10,15,16]. A similar bimodal trend is observed in canines, with a peak in dogs aged less than 3 years old, and then 80% of cases presenting in dogs aged 7 years (middle-aged) and older [9,18]. The association with OSA formation and high growth levels in puberty implicate growth and developmental factors in OSA aetiology.

Clinical presentation of canine OSA is characterised by progressive lameness, hard bony swelling, or even pathological fracture of the affected bone [5,19]. This neoplasia is very aggressive and invasive in dogs, causing local skeletal destruction and is also highly metastatic, predominantly to the lungs, with a lower frequency of spread to distant bones, regional lymph nodes, and other soft tissues [3,5]. The accepted treatment at present is a combination of radiotherapy, chemotherapy (both adjuvant and/or neo-adjuvant) and surgery [10,17,20]. For people with OSA with no concomitant metastases at diagnosis, the 5-year event-free survival stands at around 70%; however, approximately 20% of patients will exhibit metastases upon diagnosis and their 5-year event-free survival drops to 27%. In canine OSA, the 1-year survival after treatment is just 45%, whereas the median time from diagnosis to euthanasia has been reported to be as low as 111 days (range, 28 to 447 days) in one study [21]. For those dogs that survive past 1 year, over 50% will develop metastases and present with a median survival time of 243 days [7,17,22]; patient outcomes are currently limited due to the highly metastatic nature of OSA and multi-drug resistance limiting the effects of chemotherapy. Investigations into the canine model could yield new treatments specifically targeting OSA molecular pathways.

Our previous research showed that *FOXO4*, *IRF8*, and *LEF1* were differentially expressed (via RNA sequencing) in canine osteosarcoma compared to patient-matched non-tumour tissue [11,23]. FOXO4 (also known as AFX1) belongs to the forkhead box class O (FOXO) family, a group of transcription factors involved in numerous cellular processes, including development, proliferation, survival, apoptosis, metabolism, and homeostasis [24,25,26]. Post-translational changes in the FOXO group can alter their nuclear import/export, modify DNA binding affinity, and change the transcriptional activity of target genes [24]. Growth factors such as insulin and insulin-like growth factor 1 (IGF-1) can regulate the activity of FOXO4 by repressing it through the phosphoinositide—3 kinase (PI3K)/Akt signalling pathway [24,25]. Upon activation of the PI3K-AKT pathway, AKT phosphorylates FOXO proteins, impeding their localisation to the nucleus and transcriptional activity [27,28]. Without growth factors present, the FOXO transcription factors are localised in the nucleus and upregulate key genes, causing cell cycle arrest and cell death [29]. Additionally, through an interaction with p53 that represses p53-mediated apoptosis, FOXO4 has been shown to have a key role in senescent cell viability [30]. The FOXO4 protein, along with FOXO1 and FOXO3, is also important for bone development. The loss of these proteins in osteoclast progenitors can result in an increase in proliferation, bone resorption, and osteoclast formation [31]. In human OSA, a study by Chen and colleagues found that the oncogenic miRNA, miR-664, promoted cell proliferation by supressing *FOXO4* expression, suggesting that FOXO4 has a role as a tumour suppressor in osteosarcoma [32]. Consistent with this finding, FOXO4 has been reported to have reduced expression in cancer compared to non-malignant tissue and have a tumour suppressor role in several other cancer types, including colorectal, gastric, and head and neck squamous cell cancer [33,34,35]. Findings by Paik et al. [36] revealed that FOXO1, 3, and 4 are largely functionally redundant in their tumour suppressor function. Oncogenic splice variants of FOXO4 have also been reported [37].

The transcription factor, interferon regulatory factor 8 (IRF8), originally named interferon consensus sequence binding protein (ICSBP), is a member of the IRF protein family [38]. IRF8 is constitutively expressed, is IFNγ inducible, and plays key roles in multiple biological processes, including modulation of the immune response and other physiological processes (reviewed in [39]). Considering that IRF8 is expressed in hematopoietic cells, recent studies have shown that the formation of mammalian dendritic cells (DCs) requires the transcription factor IRF8 [40]. Specifically, type 1 dendritic cells (DC1s) and the tumour-associated macrophages (TAMs) require and express IRF8 [41]. This is required for the TAM ability to present cancer cell antigens, indicating that IRF8 may play a role in promoting tumour growth [41]. Like many other transcription factors, IRF8 can be dysregulated in cancer, and therefore, this study aimed to determine the protein expression of IRF8 in canine OSA.

The *LEF1* gene encodes the Lymphoid enhancer-binding factor 1 protein, which belongs to the TCF/LEF family of transcription factors known to act via the *Wnt* signalling pathway [42,43]. LEF1 is principally involved in the process of T cell, B cell, and natural killer cell development [44,45,46]; however, it also plays a role in the regulation of skin development, the hair cycle, and development of the mammary gland [47,48,49]. As an effector of the *Wnt* signalling pathway, LEF1 is also associated with regulating the cell cycle, epithelial-to-mesenchymal transition, and with tumour development and progression [50,51,52,53]. Increased LEF1 expression has been associated with carcinogenesis in many different cancer types, including melanoma, pancreatic, colorectal, and breast, as well as several myeloid and blood cancers [54,55,56,57,58,59,60].

Our previous research [11] showed that *FOXO4*, *IRF8,* and *LEF1* were differentially expressed (via RNA sequencing) in canine OSA compared to patient-matched non-tumour tissue. *FOXO4* in OSA tissue had a 1.42 Log2 fold decrease, but not significant at *p* = 0.056, compared to patient-matched non-tumour bone; *IRF8* showed a 2.33 Log2 fold decrease, *p* = 0.01, and *LEF1* exhibited a 2.2 Log2 fold increase, *p* = 0.04. Therefore, to determine the prognostic or diagnostic potential of FOXO4, IRF8, and LEF1 in canine OSA, we performed IHC on OSA specimens to determine protein expression. The present research ascertained the H-scores in the nucleus and cytoplasm (and total H-score), alongside descriptive qualitative analysis, of these proteins in OSA specimens. The present study additionally investigated tumour location (appendicular vs. axial) and sex in relation to protein expression.

## 2. Materials and Methods

### 2.1. Specimen Preparation

**Figure 1:**
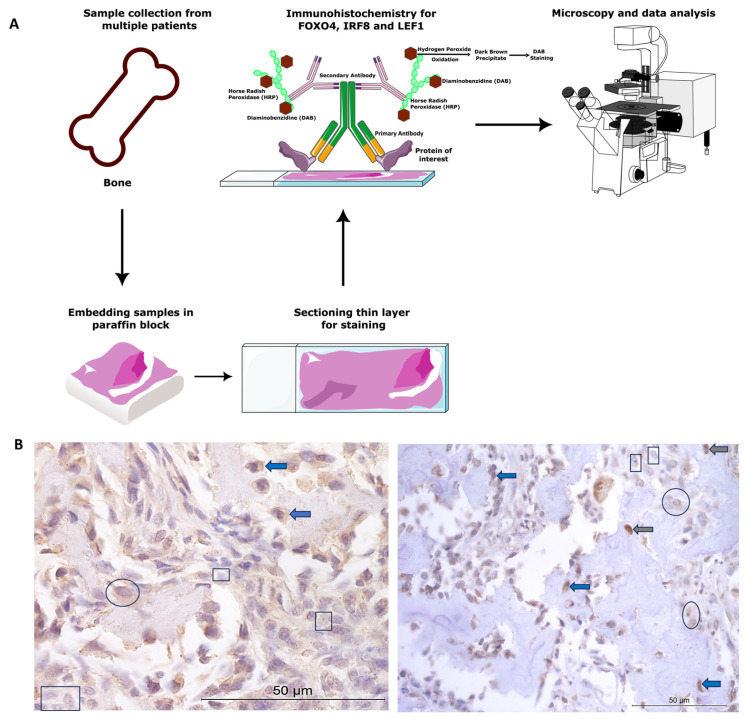
Canine osteosarcoma protein expression materials and methods. (**A**) Overview of methods. (**B**) Examples of cytoplasmic and nuclear H-scores 0, 1, 2, and 3, on IRF8 and LEF1 immunohistochemistry photomicrographs. H-score 0 (rectangles), 1+ (circles), 2+ (blue arrows), 3+ (grey arrows).

All animal tissue work in this study was approved by the ethics committee at the University of Nottingham School of Veterinary Medicine and Science. The ethics complied with national (Home Office) and international ethics procedures (permission numbers—1823 160714, 959 130925, UG 20331). The samples were from patients (Figure 1) under veterinary practice care for OSA, not related to research. A board-certified veterinary pathologist histologically confirmed the diagnosis of OSA.

Canine diagnostic OSA tissues from Rottweilers (*n* = 26) were obtained from Bridge Pathology, UK and the tissue had been formalin-fixed, paraffin-embedded. There were 13 females, 12 males, and 1 not specified. The males were between 4 and 10 years old, and the females ages ranged between 5 and 12 years old. OSA samples were from a variety of bones, including four from the head and two mammary/thoracic wall (*n* = 6 axial), and *n* = 20 from the appendicular skeleton (humerus, ulna, stifle, including the femur and tibia).

### 2.2. Immunohistochemistry and Microscopy

Paraffin-embedded samples were sectioned at 7 µm. Positive protein expression of FOXO4, IRF8, and LEF1 was visualised using a Leica Novolink Polymer Detection Kit (Leica, Wetzlar, Germany) according to manufacturer’s protocols. Primary antibodies were diluted in foetal calf serum; these included anti-FOXO4/AFX1 polyclonal unconjugated human antibody raised in rabbit (1:100 dilution, LS-C112273; LSBio, Cambridge, UK), anti-IRF8 antibody (1:500 dilution; ab28696, Abcam, Cambridge, UK), and anti-LEF1 rabbit polyclonal (1:100 dilution, GTX129186, GeneTex, Irvine, CA, USA). Negative controls were incubated in foetal calf serum only, without the primary antibody. Positive controls consisted of canine blood vessels, skeletal muscle, and nasal epithelium, as the proteins were known to be expressed in these cells and tissue types [34,61,62]. Cytoplasmic and nuclear staining was assessed following microscopy (Leica, Wetzlar, Germany), and *n* = 5 photomicrographs/specimen at 40× magnification were taken using systematic random sampling for H-scoring analysis (for methods overview, see Figure 1A).

### 2.3. H-Scoring and Statistical Analysis

H-scoring was used to semi-quantitatively analyse the IHC staining, as it is considered as one of the “gold standards” for IHC evaluation [63,64,65]. Staining intensity for each cell was designated into scores of 0, 1+, 2+, or 3+ (none, weak, moderate, strong staining signal) for each target protein (examples shown in Figure 1B). The percentage of positive staining for each score for each cell (nuclear and cytoplasmic independently) was scored to the nearest 5% for a fixed field of *n* = 5 photomicrographs per sample (*n* = 26 OSA samples) for each antibody. H-scores were calculated using the following formula: H-score = [1 × (% cells 1+) + 2 × (% cells 2+) + 3 × (% cells 3+)]. Cytoplasmic, nuclear, and total H-scores were calculated for each specimen (0–300) for each marker of interest. One double-blinded researcher undertook H-scores and established a scoring definition. Thereafter, an additional researcher scored a random 10% of the samples, to ensure concordance (intraclass correlation coefficient (ICC) > 90% for all proteins) and interpretation consistency. The mean, standard error of the mean, minimum, maximum, and range of H-scores were tabulated and plotted for FOXO4, LEF1, and IRF8 to demonstrate score distributions and staining intensities. Additionally, representative staining classifications were demonstrated as benchmarks. The H-score low/moderate/high classifications were calculated based on the ranges for each individual antibody: FOXO4 = low ≤ 34, moderate 35–69, high ≥ 70; IRF8 low ≤ 83, moderate 84–166, high ≥ 167; and LEF1 = low ≤ 62, moderate 63–124, high ≥ 125. Statistical analysis between cytoplasmic and nuclear H-scores, male vs. female H-scores, and OSA location (appendicular vs. axial) were conducted using paired *t*-test (SPSS v26). Fisher’s exact test 2 × 3 Contingency Table was used to compare the number of specimens with low, moderate, and high H-score staining categories in both the cytoplasm and nucleus.

Qualitative data were also recorded to describe general immunohistochemical staining patterns. Specifically, the tissue structures and cell types with positive immunostaining were indicated, the general staining distribution was identified for each sample (diffuse, multifocal, focal), and in addition, the overall predominant cytoplasmic and nuclear staining intensity was described following H-scoring (absent, low, moderate, high) and the main staining location was identified (cytoplasmic or nuclear).

## 3. Results

**Figure 2:**
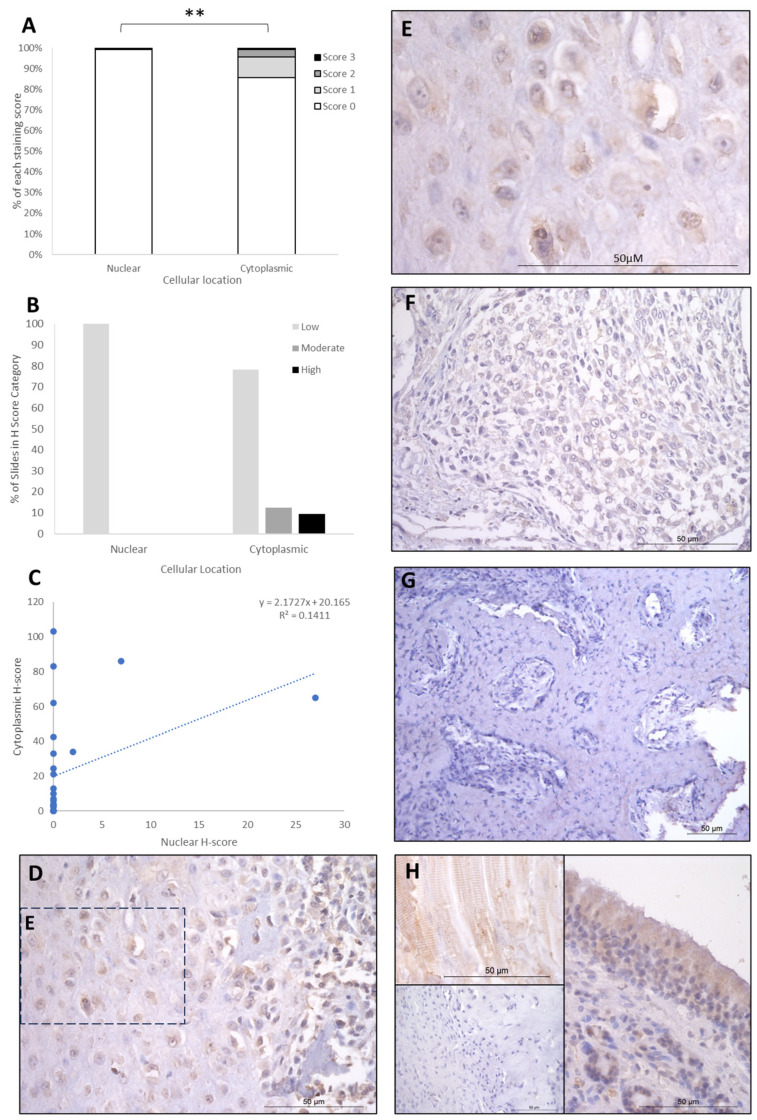
FOXO4 cytoplasmic and nuclear H-scores. (**A**) H-score (scores 0, 1, 2, and 3) distributions. (**B**) H-score low/moderate/high classifications across the cases (*p* = 0.02). (**C**) Nuclear and cytoplasmic H-score distributions and correlation. Overall, the nuclear H-scores were significantly lower than cytoplasmic (** *p* = 0.002), *n* = 26. (**D**–**G**) Immunohistochemical staining photomicrographs of canine osteosarcoma FOXO4 expression, 40× magnification. (**H**) Right-hand side: positive control nasal mucosa lined by well-differentiated pseudostratified tall columnar ciliated epithelium, inset upper left: muscle positive control, insert lower left: negative control, 40× magnification. All scale bars represent 50 µm.

The IHC staining of the three proteins is summarised in Table 1, Table 2 and Table 3. FOXO4 staining showed H-score variations between the different patients, and 8/26 specimens exhibited no nuclear or cytoplasmic staining, 15/25 expressed cytoplasmic staining only, and 3/26 exhibited both nuclear and cytoplasmic staining. Cytoplasmic H-scores for FOXO4 were low in the majority of patients (20/26; 77%), with moderate (3/26; 11.5%) and high (3/26; 11.5%) scores in the remaining dogs (Table 1). Nuclear FOXO4 scores were either absent (23/26; 88.5%) or low (3/26; 11.5%; Table 1) in all samples. Hence, a total 20 of the 26 patients (77%) exhibited both low cytoplasmic and low nuclear average scores, 3/26 (11.5%) exhibited low nuclear and moderate cytoplasmic scores, and 3/26 (11.5%) showed low nuclear and high cytoplasmic scores (Table 2). Overall, the staining was diffuse, and the nuclear H-scores were significantly lower than the cytoplasmic staining scores (*p* > 0.002, Table 3, Figure 2).

**Figure 3:**
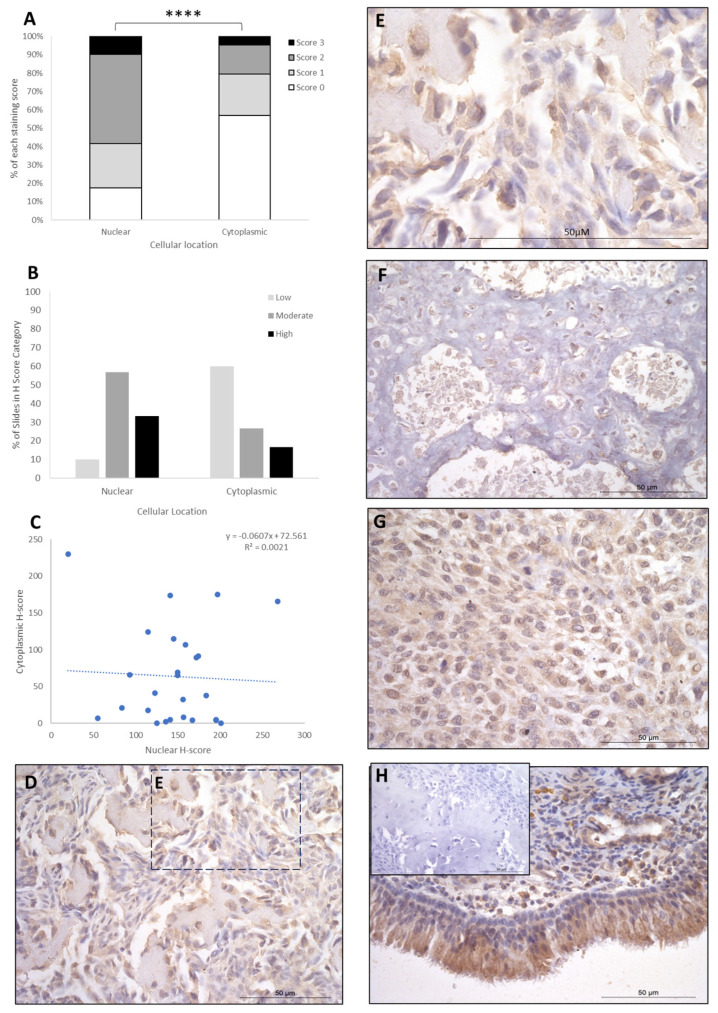
IRF8 cytoplasmic and nuclear H-scores. (**A**) H-score (scores 0, 1, 2, and 3) distributions. (**B**) H-score low/moderate/high classifications across the cases (*p* = 0.0001). (**C**) Nuclear and cytoplasmic H-score distributions and correlation. Overall, the nuclear scores were significantly higher than cytoplasmic (**** *p* = 0.0001), *n* = 26. (**D**–**G**) Immunohistochemical staining photomicrographs of canine osteosarcoma IRF8 expression, 40× magnification. (**H**) Positive control nasal mucosa lined by well-differentiated pseudostratified tall columnar ciliated epithelium, inset upper left: negative control, 40× magnification. All scale bars represent 50 µm.

IRF8 staining showed H-score variations between the different patients, and 2/26 patients exhibited nuclear staining only, whilst the remaining 24/26 had both nuclear and cytoplasmic staining. The cytoplasmic score was moderate in the majority of patients (15/26; 58%), with high (9/26; 34%) and low (2/26; 8%) scores in the remaining cases (Table 1). Nuclear scores showed 17/26 patients (65.5%) with low H scores, 6/26 (23%) at moderate, and 3/26 (12%) at high (Table 1). When assessing subcellular score combinations, low cytoplasmic scores were combined with low, moderate, and high nuclear scores in 1/26 (4%), 11/26 (42%), and 5/26 (19%) of patients, respectively (Table 2). Moderate cytoplasmic score was combined with moderate and high nuclear scores in 3/26 (11.5%) and 3/26 (11.5%), respectively (Table 2). High cytoplasmic score was combined with low, moderate, and high nuclear scores in one case each. Overall, the staining was diffuse, and the nuclear H-scores were significantly higher than the cytoplasmic staining scores (*p* > 0.0001, Table 3, Figure 3), and there was little correlation between nuclear and cytoplasmic H-scores within individual samples (Figure 3).

**Figure 4:**
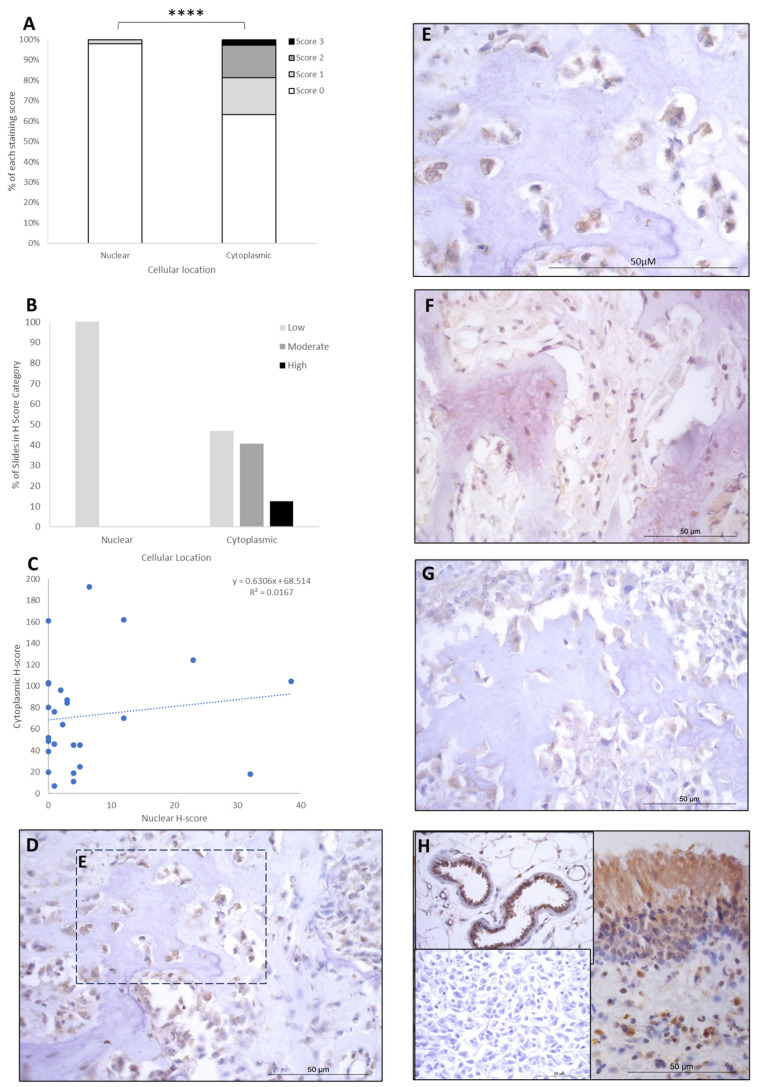
LEF1 cytoplasmic and nuclear H-scores. (**A**) H-score (scores 0, 1, 2, and 3) distributions. (**B**) H-score low/moderate/high classifications across the cases (*p* = 0.0001). (**C**) Nuclear and cytoplasmic H-score distributions and correlation. Overall, the nuclear scores were significantly lower than cytoplasmic (**** *p* = 0.0001), *n* = 26. (**D**–**G**) Immunohistochemical staining photomicrographs of canine osteosarcoma LEF1 expression, 40× magnification. (**H**) Right-hand side: positive control nasal mucosa lined by well-differentiated pseudostratified tall columnar ciliated epithelium, inset upper left: endothelial cells of vasculature positive control, inset lower left: negative control, 40× magnification. All scale bars represent 50 µm.

LEF1 staining showed H-score variations between the different patients, and 8/26 expressed nuclear staining only, while the remaining 18/26 had both nuclear and cytoplasmic staining. Cytoplasmic scores were low, moderate, and high in 12/26 (46.6%), 10/26 (38%), and 4/26 (15.5%) patients, respectively. Nuclear scores were low in 26/26 (100%) of the patients, with no cases expressing moderate or high H-scores. Low nuclear score was combined with low, moderate, and high cytoplasmic scores in 12/26 (46.5%), 10/26 (38%), and 4/26 (15.5%) of cases, respectively (Table 2). Overall, the staining was diffuse, and nuclear H-scores for LEF1 were significantly lower than the cytoplasmic staining scores (*p* > 0.0001, Table 3, Figure 4), and there was a small positive correlation between nuclear and cytoplasmic H-scores within individual samples (Figure 4). 

**Figure 5:**
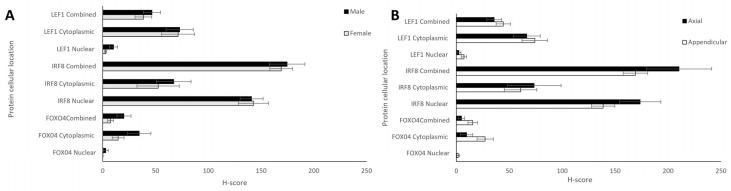
Nuclear, cytoplasmic, and combined H-scores for FOXO4, IRF8, and LEF1 by sex and anatomical location. (**A**) Males and females (*n* = 12 and 13, respectively), and (**B**) differing bone locations—appendicular and axial (*n* = 20 and 6, respectively). No statistically significant differences in sex or bone location were observed for nuclear, cytoplasmic, or total H-scores (*t*-test, *p* > 0.05).

Comparisons between the sexes and anatomical locations were also analysed for each protein. Nuclear, cytoplasmic, and combined H-scores for FOXO4, IRF8, and LEF1 in males and females (*n* = 12 and 13, respectively) showed no statistically significant differences (*t*-tests, *p* > 0.05; Figure 5A). Differing anatomical location of the bone tumours—either appendicular (*n* = 20) or axial (head + thorax; *n* = 6)—showed no significant differences for any of the proteins (*t*-test, *p* > 0.05; Figure 5B).

## 4. Discussion

The influences of genetic factors in relation to the aetiology and progression of OSA have been widely recognised, and p53 is the most frequently investigated gene in canine OSA [3]. Furthermore, there is strong documented evidence of chromosomal rearrangements, gene mutations, alterations in gene expression, alterations in microRNA expression, and DNA methylation pattern changes between human and canine OSA [66]. Another important genetic cause is the RB tumour suppressor gene, which has been associated to the development of canine OSA. In a pilot study, the expression of MET proto-oncogene was identified in the majority of the histopathological samples of seven large breed dogs with spontaneous skeletal OSA [3,67]. Importantly, canine OSA has been compared to human OSA due to similar genetic, biologic, and clinical pathological features, and it has a 14 times higher incidence rate compared to human OSA; hence, it has been used as translational medicine to understand human OSA [6,68]. A study ascribed an interesting role of miR-1 and miR-133b as biomarkers for canine OSA’s treatment and validated the high molecular homology between human and canine OSA [6]. Despite these advances, very little is known about the genetics of OSA and protein expression, the present study aimed to elucidate the expression of three proteins, FOXO4, LEF1, and IRF8, in OSA.

Previously, we have shown a 1.42 Log2 fold decrease in *FOXO4* transcripts in OSA tissue compared to patient-matched non-tumour bone [11]. In the present study, the majority of OSA specimens exhibited no nuclear protein staining of FOXO4. As little cytoplasmic expression was also observed, FOXO4 holds limited value for prognostic or diagnostic use, although expression in non-malignant bone is yet to be determined. Firstly, it is of interest that our research shows that it is expressed in bone. The Human Protein Atlas data by Santos and coauthors found FOXO4 transcript expression across numerous tissues; however, protein was detected by IHC in only the testis, placenta, heart, skeletal muscle, and smooth muscle [69], notably not bone. This discrepancy between mRNA and protein expression was not consistent with our analysis of canine OSA tissue, where the protein was expressed. There was little previously known about FOXO4 in relation to canine OSA; however, a study investigating the role of FOXO4 in human colorectal cancer found that it had a role as a tumour suppressor, as FOXO4 was downregulated in colorectal cancers when compared to the control [33]. Overexpression of FOXO4 was found to have reduced migration and in vivo metastasis of the colorectal cancer cells by regulating the colorectal cancer tumour suppressor gene adenomatous polyposis coli 2 (APC2) in the APC2/β-catenin axis; therefore, this inhibitory effect could be reversed by APC2 knockdown [33]. Another study supports this claim that FOXO4 is a tumour suppressor, as it found that its expression was decreased in human gastric cancer tissue and gastric cancer cell lines. The upregulation of this protein inhibited tumour growth and progression, whereas downregulation of this protein promoted tumour growth and progression [34]. Therefore, although FOXO4 expression was not expected in bone, its expression and its downregulation in other tumours (reviewed in [70,71]), including OSA, as shown in the present research, support its role relating to tumour suppression, and the subsequent growth and progression where it is downregulated.

We previously identified *IRF8* to be downregulated in canine OSA as compared to matched non-malignant specimens. In agreement with previous studies analysing cellular localisation of the protein in other cell types [72,73], we found positive staining in both the nuclear and cytoplasmic compartment of OSA cells, with cytoplasmic staining present at a lower level of expression. Currently, little is known about the role of IRF8 in human or canine OSA. Muhitch and coauthors [74] observed that high expression of IRF8 in combination with low levels of TAMs has a significantly better survival outcome in comparison to low levels in both TAMs and IRF8 expression metastatic renal cell carcinoma tumour. It has been shown that IRF8 promoted epithelial–mesenchymal transition (EMT)-like phenomena, cell motility, and invasion in a human OSA cell line, suggesting that it may play a role in metastasis [75]. Another group found that PD-L1 was induced by IRF8 and that in human OSA cells, PD-L1 and IRF8 were involved in growth and tumorigenicity, and that PD-L1 knockdown combined with doxorubicin treatment resulted in inhibition of cell growth [76]. Furthermore, a study identified that *IRF8* was among one of the many genes deleted in >25% of cases, according to an analysis of 28 human OSA samples [77]. Our current study presents the expression of IFR8 in canine OSA. Taken together, these studies identify a role for IRF8 in OSA, with potential as a prognostic marker, and further studies on the role and clinical relevance of IRF8 in both human and canine OSA are warranted.

Our present research shows LEF1 expression in both the cytoplasm and nucleus, and our previous work showed that *LEF1* exhibited a 2.2 Log2 fold increase in OSA samples compared to patient-matched non-tumour tissue. *LEF1* expression has also been demonstrated to be upregulated in OSA cells and patient samples compared with non-malignant osteoblasts and tissue in people [78,79,80,81]. *LEF1* has been associated with metastasis in OSA. Overexpression of *LEF1* was observed in highly metastatic OSA cell lines compared with OSA cells with low metastatic potential and, moreover, knock-out of *LEF1* resulted in significantly reduced extravasation of OSA cells to the lungs [82]. *LEF1* overexpression has also been found to abrogate the inhibitory effect of miR-34c on metastasis and chemoresistance in OSA cells [83]. It is notable that all studies investigating the role of LEF1 in OSA appear to have been conducted only in people and mice. To the best of the authors’ knowledge, the current study is the first to report on the potential role of LEF1 in canine OSA. LEF1 expression has also been shown to be downregulated in numerous cancers through promoter hypermethylation and also higher levels of *IRF8* [61,84,85]. LEF1 was initially thought to be an effective therapeutic target [86]. In 2010, two small molecule inhibitors of Wnt/beta-catenin/LEF1 signalling (CGP049090 and PKF115-584) significantly inhibited the proliferation of CLL cells in vivo [60]. A large number of other small molecule inhibitors targeting the Wnt/beta-catenin/LEF1 pathway have since been discovered, and work to improve their utility and specificity as anti-cancer treatments is ongoing [87]. Unfortunately, very few of the compounds that have shown promise in vitro and in vivo have progressed to clinical trials, and among the ones that have, many have resulted in unsatisfactory outcomes due to inhibition of the wide-ranging essential functions of this pathway in normal physiological processes [88]. The authors are not aware of any inhibitors that have advanced beyond the very early phases of clinical trials to date. LEF1 has also been purported to have utility as a prognostic biomarker, since its high expression has been significantly associated with disease progression and poorer prognosis in chronic lymphocytic leukaemia (CLL) [89], acute lymphoblastic leukaemia (ALL) [90,91], small B-cell lymphomas [86], solid pseudopapillary neoplasms and pancreatic neuroendocrine tumours [92], oesophageal squamous cell carcinoma [93], nasopharyngeal carcinoma [94], deep penetrating nevi [62], and with metastasis in colorectal cancer [95]. Proteins such as GLUT1, MMP3, and NRF2 have shown promise as canine OSA biomarkers and are involved in Wnt activation [10]. The Wnt/β-catenin/LEF1 signaling pathway has also been shown to be involved in human osteosarcoma cells and tissues via RT-qPCR, where it was indicated that LEF1 translation via degradation of DKK3 was mediated through miR-214-3p, and that cantharidin could be a prospective candidate for osteosarcoma by targeting the pathways involved [96].

Primary OSA occurs more frequently on the appendicular skeleton in around 75% of the cases; 24% on the axial skeleton; and also, very rarely, approximately 1%, in extraskeletal tissues, for instance, mammary tissue, subcutaneous tissue, spleen, bowel, liver, kidney, testicle, vagina, eye, gastric ligament, synovium, meninges, and adrenal gland [3,4]. Interestingly, this does differ in relation to dog size, with one study showing that 5% of the diagnosed large and giant breed dogs with OSA presented with axial tumours compared to 59% in small breed dogs (less of 15 kg) [97]. Appendicular canine OSA is more commonly in the metaphysis of long bones, especially of the forelimbs, with higher frequency rates affecting locations such as the proximal humerus, the distal radius, and the distal tibia in the hind limb [18,98,99]. One crucial risk factor is related to the body size, since the tumour tends to occur in major weight-bearing bones adjacent to late closing physes [13,100]. Obesity has also been postulated to promote osteoblast proliferation in the limbs, which can contribute to remodelling in response to increased stress on weight-bearing limbs [100]. In light of this, and given the fact that appendicular OSA is the most frequent presentation in large and giant breed dogs with rapid early bone growth, it is reasonable to argue that the combination of these factors can help elucidate the complex aetiopathophysiology of OSA in this species [19]. No overall differences were observed between the axial and appendicular samples within this study, but given the body of evidence relating to axial and appendicular OSA, future studies should note potential differences and consider whether the anatomical location impacts the tumours, their environment, and prognosis and treatment factors. A limitation of the present study is a relatively smaller number of samples in the axial bones, and ideally a larger number of anatomical locations should also be investigated in the future.

The males and females in this study showed no significant differences between protein expression for any of the markers investigated; these data add interesting evidence to the sex susceptibility discussions which are ongoing about OSA. There is contradictory evidence concerning sex predisposition in canine OSA [101]. Historically, males have been thought to be slightly more frequently affected than females, with a reported ratio of 1.1–1.5:1 [3]. In contrast, another study showed that females were more prone to be affected with OSA, with a ratio of 2.1:1, but this was not consistent with respect to the location of OSA [97]. Ru et al. concluded in their study there was no sex susceptibility, but neutered males and females were noted to have twice the risk for OSA compared to intact dogs for both of the sexes [12]. A retrospective case series with 744 dogs diagnosed with appendicular OSA revealed that the male-to-female ratio was 0.95:1.0, and 80.9% of the population with OSA were neutered [99]. Despite these findings, research has not found any strong evidence that sex or neuter status is a risk factor for the development of OSA in dogs. Additionally, in some of these older reports, males have been overrepresented and/or there was bias towards male dogs or neutered animals [19,98,99,100]. Nevertheless, it has been thought that endogenous sex hormones have a significant impact on OSA, and some reports go towards the consideration of a protective influence in intact dogs [99].

Understanding whether proteins can assist with diagnosis, prognosis, or treatment development is important. Several negative prognostic factors for canine OSA have been described in the literature, and these include histological grade, distant metastasis at diagnosis, age at diagnosis, large primary tumour size, high body weight, high serum alkaline phosphate (ALP) activity, proximal humeral location, prolonged duration of clinical signs before surgery, lymph node metastasis, and delayed initiation of chemotherapy following surgery [98,102,103]. Schmidt et al. confirmed in their study that tumour location and ALP activity levels are prognostic factors for both mortality and metastasis; age was only a prognostic factor for mortality [103]. Understanding the expression of these proteins in canine, and indeed human OSA, could prove beneficial for diagnosis, prognosis, and treatment development. Further studies elucidating their roles, mechanisms of action, further protein expression level studies (e.g., Western blots), and drug discovery avenues of research are recommended.

## 5. Conclusions

Unlike human medicine, diagnosis and prognosis are not presently facilitated by the use of IHC for canine OSA; however, the present work enhances the knowledge required to understand protein expression in these tissues in different OSA samples. The diagnosis of OSA can be made through a combination of signalment, clinical presentation, and radiographic findings such as lytic, proliferative, or mixed bone lesions [18,101]. Nevertheless, histopathological samples are warranted for a final diagnosis and for tumour classification based on the formation of osteoid matrix with osteoblastic, fibroblastic, chondroblastic, telangiectic, and combined subtypes [98]. The aetiology of canine OSA has not been completely established but is considered to be complex, involving physical, genetic, and molecular factors. The investigations undertaken in the present research facilitate further understanding of the roles played by these proteins within their respective molecular pathways, including altered Wnt/beta-catenin/LEF1 signalling and via differential regulation of tumour suppressor genes and proliferation, and the effects of promoter hypermethylation. This is especially important given the opportunities that advances in molecular methods for investigating canine cancer offer for diagnosis, prognosis, and treatment development [23]. Given the staining observed and their involvement in various signalling pathways, IRF8 and LEF1 are promising biomarker candidates for prognostic and diagnostic purposes and may have mechanisms which can be targeted for the development of therapeutics. A deeper understanding of the mechanisms involved in OSA represents essential contributions towards the development of novel diagnostic, prognostic, and treatment options in human and veterinary medicine contexts.

## Figures and Tables

**Table 1:** Immunohistochemistry staining overview for H-scores.

Cytoplasmic	FOXO4	IRF8	LEF1
Absent	-	2 (8%)	8 (31%)
Low	20 (77%)	-	4 (15.5%)
Moderate	3 (11.5%)	15 (58%)	10 (38%)
High	3 (11.5%)	9 (34%)	4 (15.5%)
**Nuclear**			
Absent	23 (88.5%)	-	-
Low	3 (11.5%)	17 (65.5%)	26 (100%)
Moderate	-	6 (23%)	-
High	-	3 (11.5%)	-

**Table 2:** Subcellular staining H-scores (nuclear and cytoplasmic).

	Cytoplasmic Score				
	FOXO4	Absent	Low	Moderate	High
**Nuclear score**	**Absent**	-	-	-	-
	**Low**	-	20 (77%)	3 (11.5%)	3 (11.5%)
	**Moderate**	-	-	-	-
	**High**	-	-	-	-
	**IRF8**	**Absent**	**Low**	**Moderate**	**High**
	**Absent**	-	-	-	-
	**Low**	-	1 (4%)	-	1 (4%)
	**Moderate**	-	11 (42%)	3 (11.5%)	1 (4%)
	**High**	-	5 (19%)	3 (11.5%)	1 (4%)
	**LEF1**	**Absent**	**Low**	**Moderate**	**High**
	**Absent**	-	-	-	-
	**Low**	8 (31%)	4 (15.5%)	10 (38%)	4 (15.5%)
	**Moderate**	-	-	-	-
	**High**	-	-	-	-

**Table 3:** H-scores for FOXO4, IRF8, and LEF1.

		H-Score		
Protein (*n* = 26)	Cellular Location	Mean ± SEM	*p*-Value (Cytoplasmic vs. Nuclear)	Range (Min-Max)
FOXO4	Cytoplasmic	23.17 ± 5.53	0.002	103 (0–103)
	Nuclear	1.38 ± 0.96		27 (0–27)
IRF8	Cytoplasmic	63.65 ± 12.15	0.0001	230 (0–230)
	Nuclear	146.81 ± 9.16		48 (20–68)
LEF1	Cytoplasmic	70.66 ± 8.64	0.0001	185.5 (7–192.5)
	Nuclear	6.13 ± 1.77		38.5 (0–38.5)

*n* = 26 immunostained canine OSA specimens showing inter case variation. *p* < 0.05 = significant difference.

## Data Availability

Data are available upon request from the corresponding authors.
